# Utilizing fundus images captured by two ultra-wide field imaging systems to measure diagnostic indicators and assess the grade of diabetic retinopathy

**DOI:** 10.1186/s12886-024-03835-6

**Published:** 2025-02-10

**Authors:** Yaoyao Sun, Xiuju Chen, Zongming Song, Tingting Liu, Ye Tao, Tinglan Lai, Yanru Chen, Minghan Li, Jinfeng Qu, Xiaoxin Li

**Affiliations:** 1 Department of Ophthalmology, Peking University People’s Hospital, Beijing, China; 2 Beijing Key Laboratory of Ocular Disease and Optometry Science, Beijing, China; 3 Xiamen Eye Center of Xiamen University, Xiamen, China; 4 Henan Provincial People’s Hospital, Zhengzhou, China; 5 Henan Eye Hospital, Zhengzhou, China; 6 People’s Hospital of Zhengzhou University, Zhengzhou, China; 7 People’s Hospital of Henan University, Zhengzhou, China; 8 Eye Institute of Shandong First Medical University, Eye Hospital of Shandong, Medical University (Shandong Eye Hospital), Jinan, China; 9 State Key Laboratory Cultivation Base, Shandong Provincial Key Laboratory of Ophthalmology, Jinan, China; 10 School of Ophthalmology, Shandong First Medical University, Jinan, China

**Keywords:** Diabetic retinopathy, Ultra-wide-field retinal imaging systems, Early treatment of diabetic retinopathy study diabetic retinopathy severity, International clinical diabetic retinopathy severity, Chinese clinical diagnosis and treatment guidelines for diabetic retinopathy

## Abstract

**Background:**

This study compared the effectiveness of different ultrawide field fundus imaging systems (Clarus™ 500 and Optos^®^) in diagnosing diabetic retinopathy (DR).

**Methods:**

This was a prospective, multicentre study. Retinal photographs were captured at four eye centres utilizing both the Clarus™ 500 and Optos^®^ imaging systems. The image quality and the effective retinal area were compared. Then the consistency in diagnosing the severity of DR by two distinct imaging systems was compared according to three separate grading criteria (the International Council of Ophthalmology, the Early Treatment Diabetic Retinopathy Study [ETDRS] grading diagnostic criteria, and the Chinese Clinical Diagnosis and Treatment Guidelines for Diabetic Retinopathy [2014]) were analysed.

**Results:**

A total of 113 patients, 201 eyes and 402 images were included in this study. 39 images were excluded due to the poor image quality and 363 images were finally analyzed. The Clarus™ imaging system demonstrated better image quality than the Optos^®^ imaging system (κ = 0.195). The mean effective retinal area was 490.03 ± 112.33 Disc area(DA) in the Clarus™ group and 410.41 ± 92.12 DA in the Optos^®^ group (*P* = 0.000). The κ values were 0.812 for ETDRS DR severity, 0.787 for ICO DR severity, and 0.790 for the Chinese guidelines, indicating substantial agreement between the two imaging systems. However, Clarus™ indicated higher DR severity than the Optos^®^ imaging system, with a higher detection rate of intraretinal microvascular abnormalities (IRMAs) and neovascularization.

**Conclusion:**

Clarus^™^ and Optos^®^ exhibit strong concordance in the identification of DR. Clarus^™^ offers better image quality and IRMA recognition than Optos^®^ and better identifies patients who need treatment.

**Supplementary Information:**

The online version contains supplementary material available at 10.1186/s12886-024-03835-6.

## Background

Diabetic retinopathy, a prevalent and distinct microvascular complication associated with diabetes mellitus, plays a significant role in the global health landscape [1, 2]. Notably, it is the primary preventable cause of blindness among adults of working age in numerous countries [3, 4]. Epidemiological investigations have found that the occurrence of diabetic retinopathy varies widely, ranging from 20.5 to 46.9% of the diabetic population, approximately 8% of them experiencing severe visual impairment [5, 6]. This disease poses a significant threat to public health and societal welfare, impeding productive activities and posing a substantial societal burden.

In patients with established diabetic retinopathy, the absence of symptoms is often the norm until the emergence of macular edema (ME) or proliferative diabetic retinopathy (PDR). According to evidence-based medicine reports from both national and international DR research organizations, the implementation of early screening, accurate grading and staging of the diagnosis, and rational treatment strategies can mitigate the progression of DR and significantly reduce the incidence of severe visual impairment and blindness [7, 8].

Early Treatment of Diabetic Retinopathy Study (ETDRS) 7-standard-field 35-mm colour 30° fundus images, commonly referred to as ETDRS 7-field images, have historically served as the gold standard for diagnosing and grading diabetic retinopathy (DR) [9]. The acquisition of these 7-field images requires multiple captures from the subject with dilated and well-coordinated pupils, which has restricted their widespread utilization and dissemination. This limitation is particularly prominent in DR screening settings, where lesions initially manifest in the peripheral retinal region [2, 10].

Recently, ultrawide field (UWF) imaging, encompassing a field of view of 100° or beyond, has emerged as a significant advancement in DR detection and management, surpassing traditional ETDRS 7-field imaging by revealing additional and more extensive pathologies of DR [11, 12]. Key devices capable of UWF imaging include the Optos^®^ California from Optos^®^ PLC (Dunfermline, United Kingdom) and the Clarus™ 500 from Carl Zeiss Meditec (Jena, Germany), each boasting unique design characteristics.

The Optos^®^ imaging system utilizes a scanning laser ophthalmoscope, enabling the acquisition of retinal images without the need for mydriasis. This innovative design allows for the capture of up to 200° of the retina in a single image, significantly expanding the scope of retinal examination [13, 14]. Conversely, the Clarus™ 500 fundus imaging system incorporates a combination of red, green, and blue images to produce realistic colour fundus representations. This system can capture up to 133° of the retina in a single image and 200° of the retina using two montage images (nasal and temporal periphery), ensuring high resolution and image quality [15, 16].

A comparative analysis by Hirano et al. evaluated the ability of these two UWF imaging systems to assess DR severity. Although both systems demonstrated general consistency, it is noteworthy that the study utilized a single image from the Clarus™ system rather than two-montage images. Given the widespread and efficient utilization of two-montage images from Clarus™ in recent years, further investigation is imperative to assess the imaging quality and screening effectiveness of these distinct imaging systems. To assess the comparative effectiveness of different fundus imaging systems in diagnosing and managing diabetic retinopathy (DR), we conducted a comprehensive study involving a large Chinese cohort.

## Method

This prospective, multicentre study was conducted at the Department of Ophthalmology at Peking University People’s Hospital. Participants from four ophthalmic centres were enrolled in the study (Peking University People’s Hospital, Xiamen Eye Center of Xiamen Universiy, Henan Provincial People’s Hospital and Eye Hospital of Shandong), and data were collected from January 17, 2021 to November 21, 2022. Retinal photographs were captured at four eye centres utilizing both the Clarus™ 500 and Optos^®^ imaging systems. No.

fundus fluorescein angiography was used in our study. These fundus images were uploaded to a designated reading centre for further analysis.

The inclusion criteria for the study were as follows: (1) patients with a confirmed diagnosis of diabetic retinopathy, staged from I to IV. The diagnosis grading is made according to the Chinese clinical diagnosis and treatment guidelines for diabetic retinopathy (2014). That is: mild NPDR (stage I), moderate NPDR (stage II), severe NPDR(stage III), and early proliferative PDR (stage IV) in the corresponding ICO classification criteria (excluding PDR with fibrovascular membranes and vitreous hemorrhage); (2) The proportion of patients with stage IV (PDR) must not exceed 20% of all enrolled patients; (3) patients aged ≥ 10 years at enrolment; (4) patients with relatively transparent refractive media for fundus photography; and (5) patients who understood the clinical trial and participated voluntarily, and signed an informed consent form. The exclusion criteria were as follows: (1) patients with progressive PDR, characterized by preretinal haemorrhage, fibrovascular membrane formation, or tractional retinal detachment; (2) patients who had undergone panretinal photocoagulation or ophthalmic surgery before enrolment; (3) patients with severe refractive media opacities or pupillary abnormalities that could compromise the quality of image acquisition; and (4) patients with any additional disqualifying conditions as determined by the investigators, including conditions such as nystagmus, severe strabismus, and mental abnormalities and so on that rendered the patients unable to cooperate with fundus imaging. The patient underwent fundus photography without pupillary dilatation, and their general information was subsequently recorded.

**Fig. 1:**
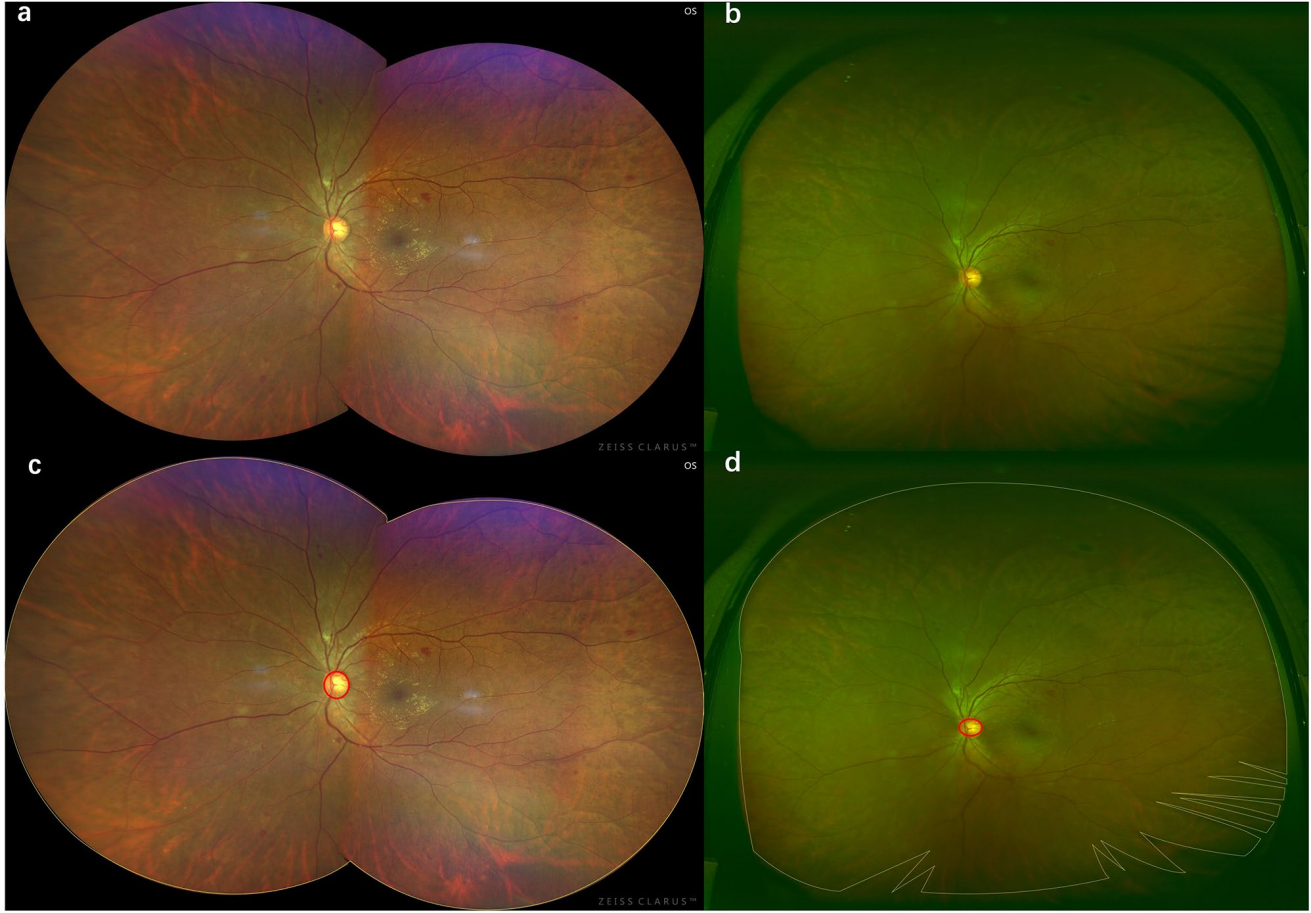
Standardized fundus images captured by two different image system. Fundus images captured by Clarus^™^ (**a**) and Optos^®^ (**b**). The yellow line indicates an obscured area and the area of visible retina were excluded and the red line indicates the optic disc. (**c** and **d**). The effective retinal area was defined as the ratio of the area (with in the yellow line) of the visible retina to the area of the disc area (DA)

Referring to the study conducted by Hirano et al., each of the fundus images was graded for the ETDRS 7-field area and all fundus images were imported into ImageJ (National Institutes of Health, Bethesda, MD) as RGB colour images with a resolution of 1024 × 1024 pixels. Using this software, the areas of the visible retina and optic nerve head were manually outlined, and the corresponding pixels were quantified [15] (Fig. 1). The effective retinal area was defined as the ratio of the area (in pixels) of the visible retina to the area (in pixels) of the disc area (DA). No adjustments were made to the brightness and contrast of the images. The evaluation of image quality is conducted using specific metrics, encompassing focus sharpness, exposure level, and artefacts (eyelashes and eyelids) pertaining to quality. An overall assessment of the image’s quality was synthesized, accounting for the collective confidence in its integrity. Two independent investigators (Y.S. and J.Q.) separately analysed the quality of the images and the total effective retinal area. Additionally, the consistency in diagnosing the severity of diabetic retinopathy (DR) using two distinct imaging systems adhering to the above diagnostic criteria was also analysed. In the event of discrepancies in the grading between the two investigators, the reading of the third investigator was deemed definitive (X.L.). The evaluation of image quality is conducted using specific metrics, encompassing focus sharpness, exposure level, and any other deficiencies pertaining to quality. Subsequently, an overall assessment of the image’s quality is synthesized, accounting for the collective confidence in its integrity.

**Fig. 2:**
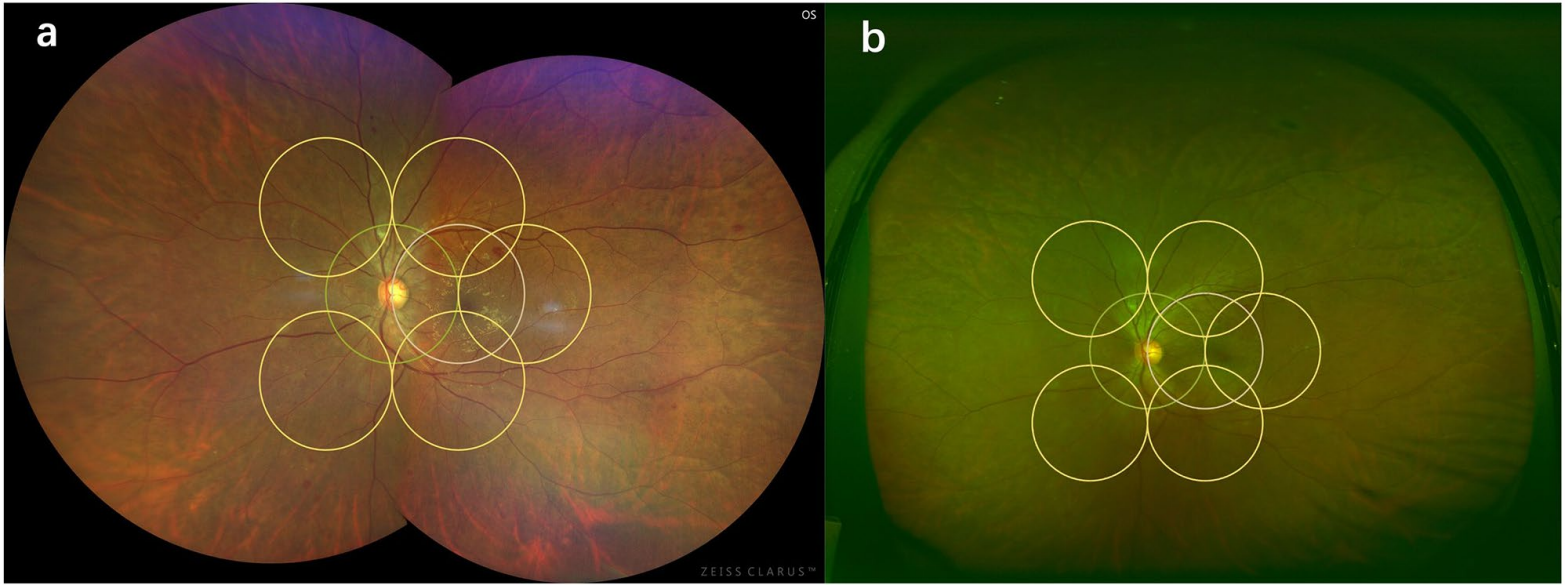
Comparison of severity of DR by Clarus^™^ (**a**) and Optos^®^ (**b**) in ETDRS 7-field area. The black line shows the area of ETDRS 7-field area and the consistency in diagnosing the severity of DR by two distinct imaging systems was compared according to three separate grading criteria

The grading of diabetic retinopathy in the study participants was determined according to multiple internationally recognized diagnostic criteria (Fig. 2). These criteria included the severity grading system established by the International Council of Ophthalmology (ICO) [17], the Early Treatment Diabetic Retinopathy Study (ETDRS) grading diagnostic criteria [9], and the Chinese clinical diagnosis and treatment guidelines for diabetic retinopathy (2014) [18]. When inconsistencies arose in the grading of fundus images between the two imaging systems, the reading centre performed both qualitative and quantitative analyses of the relevant fundus signs.

### Statistical analysis

The data were analysed using the SAS statistical package version 24.0 (SPSS Inc., Chicago, IL, USA). The paired t test or Wilcoxon signed ranks test was used to compare the effective retinal area between imaging systems, depending on whether the data conformed to a normal distribution. A two-tailed P value < 0.05 was considered significant. Agreements between two graders and the DR between two imaging systems were calculated by weighted κ statistics, as previously reported: <0.20, poor; 0.21–0.40, fair; 0.41–0.60, moderate; 0.61–0.80, substantial; and 0.81–1.00, almost perfect agreement [19].

## Results

### Demographic information

The study recruited 113 patients with 201 eyes and 402 images. However, 39 images were excluded due to inadequate image quality of either imaging system. The age distribution of the study participants ranged from 32 to 77 years, with a mean age of 58.43 ± 10.82 years. Among the participants, 68 were male (60.18%), and 45 were female (39.82%). The mean diabetes duration was 11.33 ± 6.91 years. The mean fasting blood glucose level was 7.19 ± 1.29 mmol/l (Table 1).

**Table 1:** Demographic data of the patients

Data	Number
Female, frequency (%)	45(39.82)
Age, mean (SD), y	58.43(10.82)
Mean diabetes duration, mean (SD), y	11.33(6.91)
Fasting blood glucose, mmol/L	7.19 (1.29)

The level of agreement between the two graders in assessing DR severity was found to be substantial to almost perfect. For the overall quality, the κ value was 0.788 ± 0.054. In terms of grading, for ETDRS DR severity, the κ value was 0.703 ± 0.036 according to the Optos^®^ imaging system and 0.812 ± 0.080 according to the Clarus^™^ imaging system. Similarly, for ICO DR severity, the κ value was 0.793 ± 0.037 in the Optos^®^ imaging system and 0.888 ± 0.040 in the Clarus^™^ imaging system. Finally, for Chinese guideline DR severity, the κ value was 0.791 ± 0.036 in the Optos^®^ imaging system and 0.836 ± 0.044 in the Clarus^™^ imaging system. Since all the k values are above 0.6, the two readers have a high reading agreement.

### Evaluation of the image quality and effective retinal area

Specifically, the κ values for focus sharpness, exposure level, and artefacts were 0.155 ± 0.081, 0.204 ± 0.071, and 0.217 ± 0.065, respectively. Additionally, the κ value for the comprehensive quality assessment was 0.195 ± 0.060. Notably, the Clarus^™^ imaging system demonstrated superior image quality over the Optos^®^ imaging system. The mean effective retinal areas were 490.03 ± 112.33 DA in the Clarus^™^ group and 410.41 ± 92.12 DA in the Optos^®^ group (t=-11.47, *P* = 0.000). Despite these disparities in image quality, all images remained eligible for analysis, as those failing to meet the inclusion criteria had been excluded.

The κ value was determined to be 0.812 ± 0.033 for ETDRS DR severity, 0.787 ± 0.036 for ICO DR severity, and 0.790 ± 0.036 for the Chinese guidelines. These findings indicated substantial agreement between the two imaging systems across all three DR severity grades. Specifically, in terms of the ETDRS DR grade, 17 eyes exhibited higher Clarus™ DR severity than the eyes evaluated by the Optos^®^ imaging system. Similarly, in terms of the ICO DR grade, 17 eyes demonstrated a higher DR severity in the Clarus™ system than the Optos^®^ system. Furthermore, when applying with the Chinese guidelines, 15 eyes exhibited a higher DR severity in the Clarus™ images than the Optos^®^ images.

**Fig. 3:**
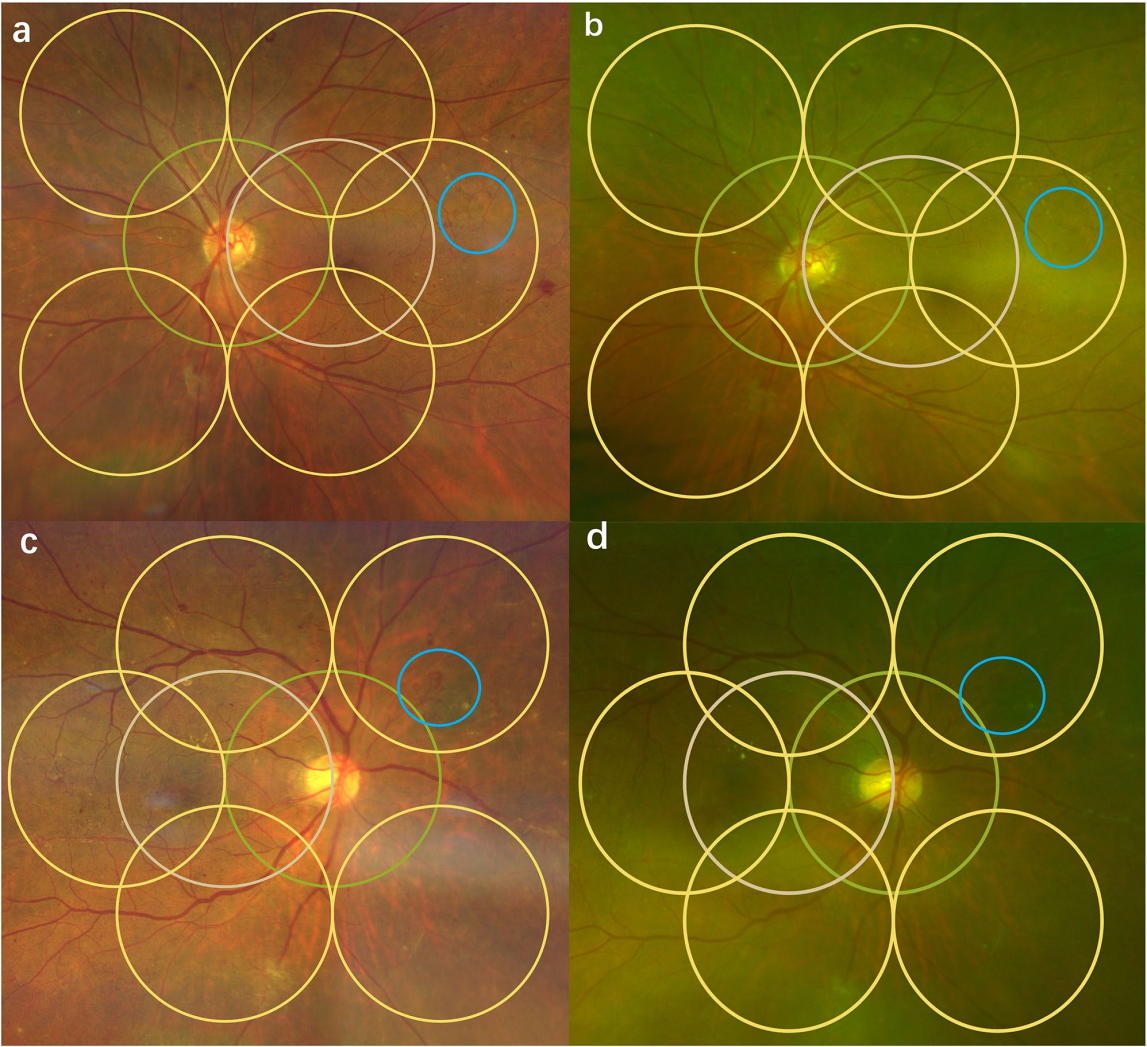
Clarus™ showed a higher detection rate of both intraretinal microvascular abnormalities (IRMA) and neovascularization (NV). Yellow circles represent the range of ETDRS 7-field area, and blue circles represent the range of lesions. Figure a is the Clarus™ image, and the blue circle shows IRMA; b is the Optos^®^ image of the same patient, and the blue circle shows that no obvious IRMA can be seen in the same location. In figure c, the Clarus™ image is shown with blue circles showing NV. D is the Optos^®^ image of the same patient, and the blue circle shows that no obvious NV can be seen in the same location

To investigate the inconsistencies in grading results between the two imaging systems for the same eye, we undertook a detailed analysis of DR retinal changes. Notably, regardless of the DR grading system employed, Clarus™ showed a higher detection rate of both intraretinal microvascular abnormalities (IRMA) (*P* = 0.038, 0.000, and 0.000 for the ETDRS, ICO, and Chinese guideline grading systems, respectively) and neovascularization (*P* = 0.000 for all three grading systems, respectively) than for the other DR retinal changes (Fig. 3).

In accordance with the American Academy of Ophthalmology (AAO) and Chinese DR guidelines regarding the appropriate timing for DR treatment, initiation of therapy is recommended once the severity of DR reaches the stage of severe non-proliferative DR (NPDR). To assess the effectiveness of the two distinct imaging systems in identifying patients requiring treatment, we calculated the proportions of patients who were deemed eligible for therapy (Table 2). Notably, regardless of the grading system utilized, the Clarus™ imaging system demonstrated a higher detection rate of patients in need of treatment than the Optos^®^ system.

**Table 2:** Effectiveness in identifying patients requiring treatment in accordance with the AAO and Chinese DR guidelines from their Optos vs. Clarus images

		Clarus™	Optos^®^	*p*
ETDRS severity	Milder than SNPDR	124	142	0.026
	SNPDR and worse	58	39	
ICO severity	Milder than SNPDR	111	137	0.003
	SNPDR and worse	71	44	
Chinese guideline severity	Milder than SNPDR	111	137	0.003
	SNPDR and worse	71	44	

SNPDR, severe non-Proliferative diabetic retinopathy

## Discussion

In this comparative analysis of two imaging systems, Clarus^™^ and Optos^®^, for the identification of DR, Clarus^™^ exhibited superior image quality and a larger effective retinal area compared to Optos^®^. Furthermore, there was substantial agreement between the two imaging systems in the grading of DR. Notably, Clarus^™^ demonstrated higher DR severity and an increased detection rate in patients requiring treatment. Based on our results, both devices can fulfil the screening requirements for DR due to their wide-angle capabilities. However, Clarus^™^ has emerged as a preferable option when making critical decisions regarding the necessity of treatment.

Since the inception of the initial wide-angle camera system in 1975 [20], the utilization of ultrawide-field imaging for visualizing the peripheral retina has ushered in a novel era in the evaluation of diabetic retinopathy. Compared to the traditional ETDRS 7-field view, ultrawide angle imaging has demonstrated a superior capacity in detecting peripheral lesions, including neovascularization, microaneurysms, venous beading, IRMA, and other manifestations, thereby leading to a more accurate and severe assessment [1, 21–23].

Optos^®^ revolutionized retinal imaging by introducing non-contact scanning laser technology that enables the capture of ultrawide field images. This technology offers a 200° field of view, encompassing approximately 82% of the retinal surface [24]. Conversely, the Zeiss Clarus™ offers single-shot wide-field images with a 133° field of view, covering approximately 570 mm^2^ (54.95%) of the retinal surface. However, by capturing two shots with an automatic montage, Clarus™ achieves a field of view of 200 × 133°, extending visualization beyond the vorticose veins to the far periphery [25]. In our study, after the removal of artefactual masking, the effective retinal area captured by the Optos^®^ imaging system was inferior to that obtained from the two montage shots of the Clarus™ imaging system. One plausible explanation for this difference lies in the technological differences between the two systems. Specifically, while Optos^®^ employs scanning laser ophthalmoscope technology, the Clarus™ imaging system utilizes a partial confocal optics system. This latter approach mitigates retinal image artefacts caused by eyelashes and eyelids. Notably, our findings contrast with those reported by Hirano, who utilized a single-shot rather than a two-shot montage with the Clarus™ imaging system. We posit that the montage of two Clarus™ shots offers a significant advantage in acquiring images for DR assessment without compromising efficiency.

Since different DR Grading systems have their own advantages, ETDRS grading is more detailed, ICO grading guideline is more operational significance and Chinese guideline is adapted to China’s national conditions, so we have analyzed different grading systems.The concordance of the ETDRS DR severity ratings between the Optos^®^ and Clarus™ images was near-perfect, regardless of the grading system employed. The ETDRS grading relies solely on fundus changes observed within the 7-field of view, whereas the ICO and Chinese grading systems incorporate a more extensive evaluation of peripheral retinal abnormalities. Consistent with Hirano’s findings, both ETDRS grading and those systems emphasizing peripheral retinal abnormalities (such as the International Clinical DR Severity Grading System Hirano used) demonstrated a high level of agreement between the two imaging systems [15]. This indicates that both systems are viable options for DR screening.

While Clarus™ excels in artefact elimination and overall image quality, Optos^®^ has its own advantages. For studies that prioritize a more extensive peripheral scoring system, Optos^®^ offers a wider field of view, capturing more information when appropriate measures are taken to avoid artefacts such as eyelashes. Additionally, Optos^®^ features a separate colour scan function, enhancing the diagnostic utility of its images. Although Optos^®^ images may be less effective than Clarus™ images for identifying microaneurysms or retinal haemorrhages, the utility of these images can be improved through the use of separate colour scan images. The integration of artificial intelligence with both imaging systems has the potential to facilitate rapid and efficient screening for diabetic retinopathy.

Specifically, in our study, certain eyes exhibited more severe DR when imaged with the Clarus™ system than when imaged with the Optos^®^ system, regardless of the grading protocol employed. Consistent with the findings of Hirato et al., we observed higher DR severity in Clarus™ images. However, when analysing the detailed retinal abnormalities associated with DR, we noted significant differences in the identification of IRMA. Although the finding was not statistically significant, our data also indicated a higher recognition rate for NV in the Clarus™ system than in the Optos^®^ system.

Despite the presence of artefacts that could obscure haemorrhages, in some patients, IRMA and NV were more apparent on Clarus™ images. Conversely, artefacts in Optos^®^ images, particularly colour artefacts, could lead to misinterpretation of haemorrhages or microaneurysms. Additionally, as Optos^®^ employs scanning laser ophthalmoscopy (SLO), laser attenuation may occur over time due to use, resulting in darkened images and compromised detailed observation.

According to the AAO and Chinese guidelines for DR, intervention is warranted when the severity of DR exceeds that of SNPDR. Given the significance of IRMA detection in diagnosing SNPDR, our findings suggest that the Clarus™ imaging system may be more effective in identifying patients requiring treatment than the Optos^®^ system. The observed differences in IRMA and NV detection between the two imaging systems further implicate IRMA and NV as crucial biomarkers for assessing DR severity. This biomarker holds particular value when comparing results across different imaging modalities and for guiding treatment decisions.

This study has some limitations. First, to maximize the sample size, our study was conducted across multiple centres, encompassing several hospitals. However, this approach introduced a potential bias in image quality, as the Optos^®^ imaging system used in different hospitals exhibited varying degrees of service time and laser attenuation. Consequently, the image quality across subcentres was not uniform. Additionally, despite adopting a centralized reading method, we acknowledged that the proficiency of the technicians in capturing images in each subcentre varied. This factor contributed to inconsistencies in the artefacts and overall image quality across the different images. Lastly, given that we are utilizing raw, uncalibrated images, the angles of the ClarusTM and Optos^®^ images do not precisely align, potentially introducing errors into the results.In the future, we aim to conduct further studies to comprehensively assess the impact of the Optos^®^ imaging system on image quality at various stages of laser decay.

## Conclusion

ClarusTM and Optos^®^ exhibit strong concordance in the identification of DR. ClarusTM offers better image quality and IRMA recognition than Optos^®^ due to the latter’s susceptibility to artefacts and laser attenuation. Consequently, ClarusTM is better at identifying patients who require treatment. As technology continues to progress, a better understanding of the unique characteristics of different machines will significantly improve the efficiency and accuracy of DR screening and treatment.

## Electronic supplementary material

Below is the link to the electronic supplementary material.

Supplementary Material 1

Supplementary Material 2

## Data Availability

The datasets generated and analysed during the current study are available from the corresponding author upon reasonable request.

## Abbreviations

DR: Diabetic retinopathy
ME: Macular oedema
PDR: Proliferative diabetic retinopathy
SNPDR: Severe non-Proliferative diabetic retinopathy
ETDRS: Early Treatment of Diabetic Retinopathy Study
DA: Disc area
UWF: Ultrawide-field
ICO: International Council of Ophthalmology
IRMA: Intraretinal microvascular abnormalities
