# Supplementary material for: Utilizing fundus images captured by two ultra-wide field imaging systems to measure diagnostic indicators and assess the grade of diabetic retinopathy
Source: BMC Ophthalmol. 2025 Feb 10;25:72. doi: 10.1186/s12886-024-03835-6 (PMC11812173; doi:10.1186/s12886-024-03835-6)
Supplement: Supplementary file 1 — Supplementary Material 1 [file 12886_2024_3835_MOESM1_ESM.docx]

| Patient No.: | | Image system: □ Clarus □Optos | OD OS |
| --- | --- | --- | --- |
| Image quality | | | |
| 1.1 Location | The distance between the middle point of the optic disc and the fovea from the image center：  □meet the standard（distance<1 PD） □poorly meet the standard（distance 1～2 PD）  □unmet the standard（distance>2 PD） □CG (Can’t Grading) | | |
| 1.2 The overall readable range of the image | □ meet the standard （ The readable range is the entire fundus image ）  □ poorly meet the standard （ The readable range of fundus images is ≥80%）  □unmet the standard （ The readable range of fundus images is < 80%） □CG | | |
| 1.3 The readable range of peripheral images | Peripheral area 3 □ meet the standard □ poorly meet the standard □ unmet the standard □CG  Peripheral area 4 □ meet the standard □ poorly meet the standard □ unmet the standard □CG  Peripheral area 5 □ meet the standard □ poorly meet the standard □ unmet the standard □CG  Peripheral area 6 □ meet the standard □ poorly meet the standard □ unmet the standard □CG  Peripheral area 7 □ meet the standard □ poorly meet the standard □ unmet the standard □CG | | |
| 1.4 Focus resolution | □meet the standard (The image is clearly visible) □poorly meet the standard（ Image barely recognition ）  □unmet the standard（ Image unrecognizable ） □CG | | |
| 1.5 Exposure | □meet the standard（ Small blood vessels and nerve fiber layers are clearly visible ）  □poorly meet the standard（ The exposure is slightly stronger or less, but the fundus lesions are clearly discernible ）  □unmet the standard（ Overexposure or underexposure ） □CG | | |
| 1.6 Quality defect | □ Eyelashes, hair or foreign body shadow □ Edge leakage or abnormal color and reflection  □Macular shadow □others（detailed description as ） | | |
| 1.7 Overall credibility | □Good □fair □poor | | |

Table S1 Image quality evaluation for signs recognition and grading diagnosis of diabetic retinopathy
