# Supplementary material for: Utilizing fundus images captured by two ultra-wide field imaging systems to measure diagnostic indicators and assess the grade of diabetic retinopathy
Source: BMC Ophthalmol. 2025 Feb 10;25:72. doi: 10.1186/s12886-024-03835-6 (PMC11812173; doi:10.1186/s12886-024-03835-6)
Supplement: Supplementary file 2 — Supplementary Material 2 [file 12886_2024_3835_MOESM2_ESM.docx]

| **ETDRS** | | | | | | | | | | |
| --- | --- | --- | --- | --- | --- | --- | --- | --- | --- | --- |
| **Grade** | zeiss | | | | | | | | | total |
|  | 0 | 1 | 2 | 3 | 4 | 5 | 6 | 7 | 8 |  |
| Number | 2 | 15 | 1 | 12 | 33 | 35 | 29 | 26 | 28 | 181 |

| **ICO guideline** | | | | | | | |
| --- | --- | --- | --- | --- | --- | --- | --- |
| **Grade** | zeiss | | | | | | total |
|  | 0 | 1 | 2 | 3 | 4 | 5 |  |
| Number | 1 | 15 | 32 | 65 | 39 | 29 | 181 |

| **Chinese guideline（2014）** | | | | | | | |
| --- | --- | --- | --- | --- | --- | --- | --- |
| **Grade** | zeiss | | | | | | total |
|  | 0 | 1 | 2 | 3 | 4 | 5 |  |
| Number | 1 | 15 | 32 | 65 | 39 | 29 | 181 |
